# Expert consensus on the evaluation and diagnosis of combat injuries of the Chinese People’s Liberation Army

**DOI:** 10.1186/s40779-018-0152-y

**Published:** 2018-02-13

**Authors:** Zhao-wen Zong, Lian-yang Zhang, Hao Qin, Si-xu Chen, Lin Zhang, Lei Yang, Xiao-xue Li, Quan-wei Bao, Dao-cheng Liu, Si-hao He, Yue Shen, Rong Zhang, Yu-feng Zhao, Xiao-zheng Zhong, representing the PLA Professional Committee and Youth Committee on Disaster Medicine, Jun-Qiang Bao, Quan-wei Bao, Lin Chen, Si-xu Chen, Guo-fu Du, Hao-jun Fan, Zhen He, Jian Huang, Li-song Huang, Jiang-tao Huo, Jian-jun Jing, Li-ping Kuai, Nan Li, Xiao-Bin Li, Xiao-dong Li, Xiao-xue Li, Guo-dong Li, Hui Liu, Tong-ting Liu, Yun-fei Niu, Zhuo-yi Qiao, Hao Qin, Ze-wo Qiu, Guo-hui Ren, Yi Shan, Yue Shen, Li-xin Shu, Jia-can Su, Bin-xiao Su, Guo-dong Wang, De-wen Wang, Jia-xing Wang, Qi Wang, Zhi-nong Wang, Wei Wu, Lei Yang, Jia-zhi Yang, Jian Yang, Yuan Yao, Bo Yu, Shuai Yue, Bin Zhang, Guan Zhang, Lian-yang Zhang, Lin Zhang, Rong Zhang, Yu-xiang Zhang, Yu-feng Zhang, Yi Zhang, Yu-feng Zhao, Sheng-hu Zhou, Zhao-wen Zong, Hong-yan Zuo

**Affiliations:** 1 State Key Laboratory of Trauma, Burn and Combined Injury, Department of Trauma Surgery, Daping Hospital, Army Medical University, Chongqing, China; 2 Special Slinic Department of Bethune Medical Profession Sergeant School, Shijiazhuang, China; 3 Research Institute of Disaster Medicine, General Hospital of Chinese People’s Armed Police Forces, Beijing, China; 4 Military Medical Training Brigade of Chinese People’s Liberation Army, Hutubi, Xinjiang, Uygur Autonomous Region China

**Keywords:** Combat injuries, Assessment, Diagnosis, Expert consensus

## Abstract

The accurate assessment and diagnosis of combat injuries are the basis for triage and treatment of combat casualties. A consensus on the assessment and diagnosis of combat injuries was made and discussed at the second annual meeting of the Professional Committee on Disaster Medicine of the Chinese People's Liberation Army (PLA). In this consensus agreement, the massive hemorrhage, airway, respiration, circulation and hypothermia (MARCH) algorithm, which is a simple triage and rapid treatment and field triage score, was recommended to assess combat casualties during the first-aid stage, whereas the abbreviated scoring method for combat casualty and the MARCH algorithm were recommended to assess combat casualties in level II facilities. In level III facilities, combined measures, including a history inquiry, thorough physical examination, laboratory examination, X-ray, and ultrasound examination, were recommended for the diagnosis of combat casualties. In addition, corresponding methods were recommended for the recognition of casualties needing massive transfusions, assessment of firearm wounds, evaluation of mangled extremities, and assessment of injury severity in this consensus.

The accurate assessment and diagnosis of injuries are fundamental for triage and treatment. Currently, no related guidelines and regulations are available for the Chinese People’s Liberation Army (PLA). Therefore, the Professional Committee and Youth Committee on Disaster Medicine of PLA edited the *Consensus on the Evaluation and Diagnosis of Combat Injuries among the PLA*. The consensus was discussed and finalized at the second annual meeting of the Professional Committee on Disaster Medicine of PLA held in August 2018 in Tianjin to provide a standard for combat injury diagnoses for the PLA.

During the editing of this consensus, the standard recommended by the Oxford Center for Evidence-based Medicine and the standards commonly used in clinical research were referenced to develop a standard for evidence evaluation and recommendation (Table 1) [1–3]. However, due to the special nature of care for combat injuries (e.g., random double-blind experiments are not available), we also integrated the Grading of Recommendations Assessment, Development and Evaluation (GRADE) system to rate the quality of the evidence and the strength of the recommendation [4]. The advantage of the GRADE standard is the ability to provide recommendations based on a comprehensive evaluation, even for cases with low-grade evidence or a lack of the high-grade evidence needed for evidence-based medicine. Therefore, the GRADE standard is suitable for the evaluation of combat injuries and diagnostic recommendations. In this consensus, each recommendation is provided with the evidence and recommendation grades in an “evidence grade/recommendation grade” format.

**Table 1:** Grading standard for the evidence evaluation and recommendations in the current consensus [1–4]

Grading	Grading standard
Grading for evidence Evaluation	Grading standard for evidence evaluation
Grade A	Randomized controlled clinical trials or meta-analyses based on such trials using proper methods.
Grade B	Randomized controlled clinical trials, partially randomized controlled clinical trials, or meta-analyses based on such trials using methods with some insufficiencies.
Grade C	Retrospective case studies or meta-analyses based on such studies.
Grade D	Non-controlled studies (such as case reports), expert opinions, or evidence from basic medical research.
Grading for recommendations	Grading standard for recommendations
Type I	A medical measure is proven and/or commonly agreed upon to be beneficial, useful, and effective.
Type II	The effectiveness of a medical measure is still controversial.
IIa	The evidence and/or perspective tends to be useful and effective.
IIb	The evidence and/or perspective has not been proven useful and effective.
Type III	A medical measure is proven and/or commonly agreed upon to be ineffective, may be harmful in some cases, and hence is not recommended.

## Overview

**Consensus 1:** Assessment and diagnosis of combat injuries are fundamental for triage, treatment, and evacuation (Grade C/Type I).

**Consensus 2:** Due to the special nature of combat injuries, only rapid assessment and judgment can be performed in combat. Such assessments and judgements require further correction and completion in the treatment steps (Grade D/Type I).

Accurate judgement, assessment, and diagnosis of injuries in combat are the bases and foundations for triage, treatment implementation, and evacuation [5]. Different from day-to-day medical practices, special considerations must be applied for the evaluation and diagnosis of combat injuries [5]. First, limited auxiliary examination equipments are available in combat situations. Moreover, the condition of the battle environment does not allow detailed physical examinations and auxiliary examinations. Therefore, most often only preliminary evaluations can be performed. More detailed diagnoses can only be achieved in further treatment steps in early-stage treatment facilities. Second, injuries that occur in battle are usually severe, and medical professionals are more likely to see a high number of casualties at once. Therefore, a simple, rapid, and effective method is needed for injury evaluation, especially for the battlefield first aid stages. Third, the key point of injury assessment and diagnosis is different for the different treatment steps. Therefore, the injury assessment method is also different. Fourth, combat injury assessment is a continuous process that requires further correction and complement in subsequent treatment steps.

Because the evaluation method in the specialized hospitals is mostly the same as that implemented under normal conditions, this consensus only introduces the combat injury assessment methods currently applied in battlefield first aid, emergency treatment, and the early treatment stages for the PLA. Notably, the current treatment steps were adjusted in the *2016 Rules for Combat Casualty Care*, which will be published soon. In these recommendations, the medical treatments for the emergency treatment stage in the current system are split and integrated into the battlefield first aid and early treatment stages. After publication of the new regulations, this consensus will be adjusted accordingly.

Due to the special requirements for the diagnosis for injuries caused by chemical, biological, and nuclear weapons or new-concept weapons, this consensus does not include the assessment and diagnostic methods for these types of injuries.

## Injury assessment on the battlefield

**Consensus 3:** Battlefield injury assessment should be performed without threat of attack from opponents (Grade D/Type I).

**Consensus 4:** The sequential evaluation method for Massive Hemorrhage, Airway, Respiration, Circulation and Hypothermia(MARCH) is recommended for rapid injury evaluation. This method sequentially evaluates whether a lethal hemorrhage, airway blockage, tension and/or open pneumothorax, circulatory function disorder, or hypothermia has occurred. Additionally, treatment for lethal injuries should be performed while assessing the injury (Grade B/Type IIa).

**Consensus 5:** The field triage score (FTS) or simple triage and rapid treatment (START) are recommended for assessment and confirmation of the injury condition and to determine the medical and evacuation priority (Grade B/Type IIa).

**Consensus 6:** The following MARCH and START assessment results should be combined to provide red, yellow, blue, or black identifiers for the wounded to wear; these colors represent emergency treatment, priority treatment, regular treatment, and awaiting treatment, respectively (Grade D/Type IIa).

The most important prerequisite for battlefield injury assessment is safety on the battlefield. The assessment should be performed without threat of attack from opponents or with protection from an effective cover. Battlefield injury assessment should highlight the need to be rapid and accurate. In other words, the injury condition should be confirmed, and the treatment should be implemented as soon as possible. The MARCH sequence should be employed for rapid injury assessment. The FTS or START method can be implemented to evaluate and confirm the injury condition and to determine the medical and evacuation priority.

### MARCH sequence for the assessment of life-threatening injuries

In day-to-day injury treatment, the ABCDE sequence is recommended for in-field injury assessment. However, based on the experiences of the US Army during the Afghanistan and Iraq Wars, the injury assessment sequence adopted in-field should be adjusted to suit the needs in battle. The MARCH sequence is recommended for injury assessment [6]. “M (massive hemorrhage)” refers to the presence of a lethal massive hemorrhage, “A (airway)” refers to the presence of airway blockage, “R (respiration)” refers to the presence of an tension or an open pneumothorax, “C (circulation)” refers to the presence of hemorrhagic shock, and “H (hypothermia)” refers to the presence of hypothermia. This sequence was based on the finding that massive hemorrhage, airway blockage, and tension pneumothorax were the major causes of preventable death during the Afghanistan and Iraq Wars [7, 8]. Massive hemorrhage was the primary cause of preventable death and accounted for more than 80% of the total preventable deaths, whereas airway blockages and respiratory disorder accounted for only 10%-15%%. Therefore, the US Army recommended assessing massive hemorrhage as the top priority. Different from ABCDE, a check for hypothermia was added to the MARCH sequence. Hypothermia is one of the factors in the “Triangle of death” in trauma patients, which greatly influences the prognosis of the wounded [9]. The “Triangle of death” refers to the presence of metabolic acidosis, hypothermia, and coagulopathy in severely injured patients. The fundamental reason is massive hemorrhage with the need of a large amount of blood and fluid infusion, which causes disorders in the internal physiological milieu. The presence of the “Triangle of death” suggests that the wounded patient is in an extreme physical condition with an extremely high risk of death.

### START method

The START method has been widely applied for the assessment of injuries during disaster rescue. This method has also been adopted by the military of the United States, the United Kingdom, Australia, and some North Atlantic Treaty Organization (NATO) countries during battles for injury triage [10, 11]. This assessment method can be briefly explained as the “30–2-can-do” principle, where “30” refers to whether the respiratory rate is more than 30 breaths/min, “2” refers to whether the capillary refill time is more than 2 s, and “can-do” refers to whether the wounded can follow directions and walk. Through the assessment, the wounded are divided into 4 categories (emergency treatment, priority treatment, regular treatment, and awaiting treatment); the wounded are provided with red, yellow, blue, and black identifiers, respectively, and are treated and evacuated following the indicated priority.

The current identifiers adopted by the PLA are composed of 5 colors, with red representing bleeding, white representing fracture, black representing contagious diseases, blue representing radiation-caused injuries, and yellow representing intoxication. This identifier system currently adopted by the PLA is different from that employed in the START method. To align with the international method, the colors red, yellow, blue, and black are employed as identifiers representing emergency treatment, priority treatment, regular treatment, and awaiting treatment, respectively, in the soon-to-be-published *2016 Rules for Combat Casualty Care*.

### FTS method

Eastridge et al. [12] recommended use of the FTS method for battlefield injury assessment due to its simplicity in implementation. The evaluation indicators for the FTS method include the radial artery pulse and the Glasgow coma scale-motion scale. If the radial artery pulse is weakened/disappears or the motion scale is less than 6, then the score is 0. If the radial artery pulse is normal or the motion scale is normal, then the score is 1. The sum of the scores from the two indicators can be 0, 1, or 2 points. The comparison analysis showed that FTS was simple and practicable. FTS represents a simple triage method for rescuers in the frontline of the battlefield. Additionally, mortality and the duration of hospitalization can be predicted.

## Injury assessment for emergency treatment staging

**Consensus 7:** The MARCH assessment sequence is recommended for emergency care institutions to determine injuries that require immediate attention (Grade B/Type IIa).

**Consensus 8:** An abbreviated scoring method for combat injury or the START method is recommended to determine the priority for admission, treatment, and evacuation for emergency care institutions (Grade C/Type IIa).

The objective of injury assessment in the emergency treatment stage is to recognize injuries that require immediate attention and then to admit, treat, and evacuate patients according to the priority. The MARCH sequence can also be implemented for injury evaluation to recognize injuries that require emergency treatment; examples of such treatments and injuries include massive hemorrhage, airway support, and main artery injuries that require temporary diffluence. The abbreviated scoring method for combat injury adopted by the PLA [13] and the START method are more practicable and can serve as the basis of triage for treatment and evacuation. The prehospital triage instruments commonly utilized under normal conditions, such as the circulation, respiration, abdominal/thoracic, motor, and speech (CRAMS) scoring system [14, 15], the trauma score (TS), and the triage-revised trauma score (RTS) [16], are still useful in war time; the CRAMS scoring system is especially applicable [5]. The priority for admitting, treating, and evacuating the wounded is determined based on these assessments. Then, the medical staff fills out a medical tag based on the evaluation results and records the injury and treatment conditions for each of the wounded. The START method is implemented as previously described. For the CRAMS method, please refer to the relevant literature [14, 15]. In the following sections, only the abbreviated scoring method for combat injury is introduced.

### Abbreviated scoring method for combat injury

The indicators monitored in the abbreviated combat injury scoring system are the respiratory rate, systolic pressure, and consciousness, for a total score of 12 points. Patients with injuries resulting in scores less than or equal to 5 points are considered critically injured, those given scores of 6–9 points are considered severely injured, wounded members given 10–11 points are considered moderately injured, and those with 12 points are considered mildly injured (Table 2) [14].

**Table 2:** The abbreviated scoring method for combat injuries [13]

Indicator Score	Respiratory Rate (per min)	Systolic Pressure (mmHg)	Glasgow Coma Scale
4	10–29	> 89	13–15
3	> 29	76–89	9–12
2	6–9	50–75	6–8
1	1–5	1–49	4–5
0	0	< 1	3

## Injury assessment and diagnosis for early treatment institutions

**Consensus 9:** In early treatment institutions, a more accurate diagnosis can be made using the combination of the medical history, physical examination, laboratory examination, and imaging examination (Grade D/Type IIa).

**Consensus 10:** The AMPLE method (A. allergies; M. currently used medications; P. past illness/pregnancy; L. the last meal; E. events/environments related to the injury) is recommended for gathering the medical history. Information on allergies, medications, and medical history as well as pregnancy, food intake, and cause of injury should be obtained (Grade B/Type IIa).

**Consensus 11:** In early treatment institutions, implementation of the “CRASH PLAN” sequence is recommended for physical examination. A biopsy can help determine whether the thoracic or abdominal organs are injured (Grade C/Type IIa).

**Consensus 12:** Considering the limited equipment available in early treatment institutions, we recommend the use of a complete blood count, biochemical exam, blood gas test, and coagulation test to achieve comprehensive assessment of the wounded (Grade C/Type IIa).

**Consensus 13:** Use of B-ultrasonography is recommended to assist in the diagnosis of abdominal hemorrhage, pneumothorax or hemothorax, blood vessel or nerve injuries, and mild head trauma (Grade B/Type I).

**Consensus 14:** The diagnosis performed in the early treatment institution should at least include the followings: (1) diagnosis of the injury location and type; (2) diagnosis of the injury and complications; (3) diagnosis of comorbidities; and (4) diagnosis of the injury severity (Grade C/Type IIa).

**Consensus 15:** In early treatment institutions, multiple indicators should be monitored to perform a comprehensive assessment and to recognize patients who require damage control surgeries. Currently, the indicators of the need for damage control surgery include the following: (1) severe organ injuries accompany by main blood vessel injuries; (2) severe and multiple injuries; (3) massive blood loss; (4) the presence of hypothermia, acidosis, and coagulopathy; and (5) the abovementioned indicators at their thresholds when the expected time-until-surgery is more than 90 min (Grade B/Type IIa).

**Consensus 16:** By combining the injury mechanism and laboratory test results, patients who need massive blood infusions can be predicted and recognized to improve the success rate in rescuing the wounded. Current evidence suggests that the possibility of need-for-massive infusion is high when the following conditions are observed: massive hemorrhage that is difficult to control; traumatic amputation of an extremity; severe pelvic fractures; abnormal laboratory test results, such as a base deficit greater than 6 mmol/L or hemoglobin less than 110 g/L (Grade A/Type I).

**Consensus 17:** The Red Cross Classification of War Wounds is recommended for the assessment of the type and severity of combat injuries and serves as a guide for trauma treatments (Grade B/Type IIa).

**Consensus 18:** Information on the injury mechanism, clinical manifestation, extremity injury severity, Doppler ultrasound, and CT angiography should be integrated to determine the level of limb injury and guide the treatment (Grade B/Type IIa).

**Consensus 19:** Assessment of the injury severity in early treatment institutions benefits for triage, treatment plan selection, and prognostic estimations while providing a basis for clinical research. The military abbreviated injury scale (AIS)/injury severity score (ISS) [(mAIS/ISS)-2005] or the Military Combat Injury Scale (MCIS) is recommended for the injury severity assessment (Grade B/Type IIa).

The objectives of the injury assessment performed in early treatment institutions are as follows. (1) The priority for admission, treatment, and evacuation should be confirmed through assessment. For this purpose, AIS or another commonly implemented scoring system can be employed for rapid scoring and classification. The assessment method and principle are the same as previously described. (2) The early treatment institutions of the PLA are equipped to perform a B-mode ultrasound, X-ray examination, complete blood count, and blood biochemical tests. A more accurate diagnosis of the wounded can be achieved by combining the information for the clinical manifestation, physical signs, and auxiliary examinations, which then guide the treatments given in the early treatment institutions. (3) Based on the special needs in battles, assessment of firearm injuries, the extremity injury severity score, and evaluation of need for a high infusion volume should be performed to assist treatment for the special injuries received in battles. (4) The injury severity should be assessed to guide the treatment, predict the prognosis, and improve the treatment plan.

### The combat injury diagnosis for early treatment institutions

#### Medical and injury history

Understanding the process and cause of injuries is beneficial for determining potential injuries. After effectively treating life-threatening conditions, the “AMPLE” sequence should be followed to learn the medical and injury history of the wounded. The meaning of AMPLE is as follows: A refers to allergies; M refers to currently used medications; P refers to past illness/pregnancy; L refers to the last meal; and E refers to events/environments related to the injury [17].

#### Physical examination

Injuries that occur in battle tend to be severe, with a high probability of multiple injuries. Therefore, a detailed physical examination is needed to prevent misdiagnosis. Currently, the “CRASH PLAN” sequence is recommended for a focused physical examination. The meaning of the acronym is as follows: “C (cardiac)” refers to the examination for heart or circulatory system injuries; “R (respiration)” refers to the examination for chest or respiratory system injuries; “A (abdomen)” refers to the examination for abdominal injuries; “S (spine)” refers to the examination for spinal column or spinal cord injuries; “H (head and hypothermia)” refers to the examination for head injuries and hypothermia; “P (pelvis)” refers to the examination for pelvic injuries; “L (limb)” refers to the examination for limb injuries; “A (arteries)” refers to the examination for arterial injuries; and “N (nerve)” refers to the examination for nerve injuries.

Simple examinations, such as biopsies, can greatly help the determination of the presence of thoracic or abdominal organ injuries and are specifically suitable for conditions where treatments are limited (e.g., during battles). The presence of pneumothorax or hemothorax found by thoracocentesis indicates injuries of the lungs and pleura. The bile, gas, pus, or coagulated blood found by abdominocentesis indicates injuries of the blood vessels or organs. When obtaining a diagnosis is difficult, diagnostic peritoneal lavage may be useful.

#### Laboratory tests

The following laboratory tests can be performed in the early treatment institutions of the PLA:

1. **Complete blood count.** The critical indicators that should be monitored for the wounded are as follows: (1) the amount of hemoglobin and hematocrit; observation of less than 70 g/L of hemoglobin or less than 18% hematocrit suggests massive and acute hemorrhage and a need for a red blood cell infusion and (2) white blood cell counts and neutrophil ratios; observations of white blood cell counts greater than 10 × 10^9^/L and neutrophil ratios higher than 80% are indicators of combat injury-caused systemic inflammatory response syndrome.
2. **Kidney function and blood biochemical tests.** Various forms of water and electrolyte disorders are found in severely injured patients. Among these disorders, conditions such as hypoglycemia and hypokalemia are most commonly seen. A prompt blood biochemical test is necessary, and repeats of the test are needed to monitor for electrolyte disorders and to determine the type of disorder observed.
3. **Blood gas analysis.** Disturbances of the acid-base balance are significant in combat injuries. The presence of the “Triangle of Death”, which consists of acidosis, coagulopathy, and hypothermia, is the major cause of death for those wounded in battles [16]. Additionally, for patients with hemorrhagic shock, a rapid decrease in lactic acid to 2 mmol/L is an indicator for the evaluation of whether fluid resuscitation will be successful. Studies have shown that the prognosis for the wounded patient is worse with an extended duration with a high lactic acid level [18, 19].
4. **Coagulation function test.** Severe combat injuries can cause hemorrhagic shock and severe tissue damage due to the loss of coagulation factors via massive hemorrhage. Activating coagulation functions, fibrinolysis, and anti-coagulation lead to coagulation function disorder that can severely impact the patient prognosis. Therefore, blood biochemicals should be monitored to assess the coagulation functions. Typically, the condition of the coagulation function can be determined by indices, such as the prothrombin time, international normalized ratio, and D-dimer [20, 21]. Moreover, the coagulation function can be monitored using thromboelastography, which is more accurate than regular coagulation tests. Thromboelastography also allows dynamic monitoring of thrombus formation and abnormal conditions in platelet function, fibrinogen, and fibrinolysis. Currently, the early treatment institutions of the PLA are not equipped with thromboelastography; considering its significance in evaluating coagulation functions, thromboelastography will most likely be installed in early treatment institutions in the near future [22].

#### Imaging examination

1. **X-ray.** X-ray imaging assists in the diagnosis of fractures, dislocations, pneumothorax, hemothorax, and free gas in the chest and abdomen; thus, this method should be considered a basic examination for trauma patients. However, excessive movement can further worsen the injury, and thus, patients should not be moved much simply for X-ray examination.
2. **Computed tomography (CT).** Helical CT is applicable for the examination of trauma patients; this method is advantageous in examining chest and abdominal trauma, complex fractures, head or brain injuries, and multiple injuries. During the Iraq and Afghanistan Wars, the level III military treatment facilities of the United States were equipped with CT examination instruments. Therefore, the accuracy of the diagnosis of head and brain trauma, blood vessel injuries, and complex chest and abdominal injuries was increased, and the probability of disability was reduced [23, 24]. The early treatment institutions of the PLA have not been provided with CT examination instruments at present. However, considering the critical functions of CT, these instruments should be installed in the early treatment institutions in the near future.
3. **B-mode ultrasound.** B-mode ultrasound provides the advantages of being convenient, rapid, non-invasive, and easily portable. Bedside examination is possible when using ultrasound, which reduces further injury of the patient due to movements [25–27]. The United States military also provides B-mode ultrasound to assist with the diagnosis of tension pneumothorax during battlefield emergency rescue. For US level III military treatment facilities, B-mode ultrasound is also advantageous in improving the accuracy of the diagnosis of nerve injuries, abdominal injuries, and mild head and brain injuries [26–30].

In summary, the combination of the patient medical history, physical examination, and auxiliary examination allows an accurate diagnosis. Typically, the diagnosis for the wounded should at least include the followings [31]: (1) the injury diagnosis should be specific and expressed in the form of the injury location and injury type; (2) diagnosis of injury complications, which should include the diagnosis of pathological and physiological conditions; (3) diagnosis of comorbidities; and (4) assessment of the injury severity. Additionally, a critical objective of injury assessment in early treatment institution is the determination of the need to perform injury control surgeries. Currently, the indications for the need for surgical injury control include the following [32]: (1) severe organ injury accompanied by injuries in major vessels; (2) multiple injuries with severity scores ≥25; (3) massive hemorrhage; (4) the presence of hypothermia, acidosis, and coagulopathy; and (5) the abovementioned indices at their thresholds with an expected time-until-surgery > 90 min.

### Prediction of massive hemorrhage

Severely wounded patients typically experience massive hemorrhage. Therefore, active resuscitation using blood components is necessary [33, 34]. However, patients often require the implementation of injury control resuscitation. Thus, accurate recognition of these patients not only helps conserving the combat injury treatment resources but also improves the survival rates of the patients. During the Iraq and Afghanistan Wars, some teams of the US Army performed random, double-blind, and multicenter studies. Various anatomical, physiological, and laboratory indicators were shown to predict patients who might require large infusion volumes. These indicators included the following [34–37]: (1) injury mechanisms including uncontrollable massive hemorrhage in the torso, axilla, or inguinal region as well as traumatic amputation of extremities, a large-area perineum wound, or a severe pelvis fracture and (2) presentation of any of the following test results: systolic pressure ≤ 90 mmHg and or heart rate ≥ 120 times/min, abdominal ultrasound indicating a massive hemorrhage, laboratory test indicating a base deficit > 6 mmol/L, an international normalized ratio ≥ 1.5, hemoglobin less than 110 g/L, hematocrit < 32%, and a pH < 7.25.

### Evaluation of firearm injuries

A simple and rapid determination for the severity of firearm injuries can guide treatments, such as debridement [38, 39]. The Red Cross Classification of War Wounds can be applied in battlefield triage and has various advantages, such as being simple, practical, and accurate. The classification is based on 6 indicators (injury surface entry/exit diameter, presences of a cavity, fracture, injuries in major organs, such as the brain, and metal residues). These indicators are monitored for the comprehensive assessment of the type and severity of firearm injuries and provide information for wound treatment (Table 3) [38, 39].

**Table 3:** Red Cross classification of war wounds [38, 39]

Wound classification	Types of wounds			
	Soft Tissue injury (type ST)	Fracture type (type F)	Critical organ injury (type V)	Critical organ injury in combination with fractures (type VF)
Level I	Small, simple wound	1F	1V	1VF
Level II	2ST	2F	2V	2VF
Level III	3ST	3F	3V	Large wound that is life-threatening or damaging limb functions

Note: Levels I, II, and III indicate the severity of the injury and the estimates of the impact. Level I wounds are those with entry and exit wound diameters less than 10 cm, a cavity diameter less than 2 finger widths, no fracture, or a simple fracture. Level II wounds are those with entry and exit wound diameters less than 10 cm but a cavity diameter is greater than 2 finger widths or with complicated by fracture. Level III wounds are those with entry and exit wound diameters and a cavity diameter greater than 2 finger widths or complicated by fracture

### Assessment for traumatic amputation

Incidences of limb injuries were highest during wars throughout history [37]. In modern battles, the ratio of wounds caused by explosions is increasing, which has led to increases in traumatic amputation [40, 41]. Currently, no index is available to guide the determination for limb amputation or limb salvage. Generally, amputation should be considered when the patient presents with conditions such as major artery damage, main nerve injuries, a wide range of muscle soft tissue injuries, high lactic acid, or an over-extended warm ischemia time [40–42]. Additionally, the scale for limb damage severity can assist in the determination for amputation. Based on the experience of the United States military during the Afghanistan and Iraq Wars, the combination of symptoms, the limb damage severity score, Doppler ultrasound, and CT angiography assessment of vessel injuries can improve accuracy in the assessment for traumatic amputation [41–43].

### Assessment of injury severity

Assessing injury severity in the early treatment institution is advantageous for triage, treatment selection, and prognostic prediction. Moreover, the assessment provides the basis for future clinical research [5, 44]. The assessment process in battles is mostly the same as the process in day-to-day applications. However, when implementing the civilian AIS/ISS for combat wound assessment, disadvantages, such as under-estimating the severity of the combat wound and insufficient evaluation of penetrating wounds, are observed. Therefore, the United States military published a severity assessment method tailored for combat injuries.

#### Military AIS/ISS-2005

Combat wounds are different from those seen in day-to-day life. Wounds seen in normal settings tend to be blunt injuries, whereas penetrating wounds are more common in battles. The AIS/ISS provide few descriptions of penetrating wounds. Therefore, application of these scales and scoring systems is insufficient. Additionally, the civilian AIS/ISS do not give enough weight to burns and soft tissue damage. Therefore, the United States military edited the Military AIS-2005 (mAIS-2005). In the mAIS-2005, the division of levels is the same as that in the AIS, and the score also ranges from 1 to 6 points. Moreover, in the mISS, the sum of the squares of the scores from the 3 most severe wounds is calculated to give a total from 1 to 75 points, which is same as the civilian scoring system. Changes made in the mISS include the following: (1) emphasis on the higher severity of combat injuries compared to day-to-day injuries; the injury severity levels are increased, with most injuries (92%) increasing by 1 point and some increasing by 2 points, which suggests that these injury types can lead to death in battle, and (2) an increase in the codes for injuries seen in battles, such as chest impact, that are not included in the civilian AIS [45–47]. In 2016, the Joint Theater Trauma System and the expert group from the US Army Institute of Surgical Research analyzed the scores of patients in the combat casualty registry database. They compared the sensitivity and specificity of the military ISS and civilian-use ISS in assessing the injury severity. The mISS performed better that the ISS in predicting the mortality of the wounded [48].

#### Indexing combat injuries

The military AIS-2005 better described penetrating combat wounds but lacked descriptions for the special nature and the complexity of combat wounds. Therefore, in November 2008, a new Military Combat Injury Scale (MSCI) was developed at the meeting of the Institute of Surgical Research of the United States Army located in San Antonio, TX, USA [49]. The developed scale is composed of work in the following 4 sections. (1) Combat-specific body regions were defined. The MSCI used the four anatomic regions of the body with the addition of one region to account for multiple regions. Region A covers the head and neck. Region B covers the torso, including the chest, abdomen, pelvic girdle, junctional areas, such as the axilla and groin, clavicle bones, and shoulder blades. Region C covers the upper extremities. Region D covers the lower extremities. Region E is the multiple region, which indicates that the injury does not belong to any of the abovementioned 4 regions. (2) Combat-relevant severity scales were defined. (3) Descriptors were added for stress-response injuries and head and brain tissue damage. (4) A MCIS coding scheme was generated for specific injuries or injury groups. The experts developed a coding system that contained 5 digits for a total of 269 types of injuries. The 1st and 2nd digits describe the severity and region of the injury. The 3rd digit describes the type of injury. The 4th and 5th digits describe specific injuries in combination with the first 3 digits. Among the total of 269 codes, 51 (19%) are not covered in the AIS 2005 and 2008 editions. This coding scheme is simple and practical and allows specific description and distinction of injuries. Compared to the AIS-2008, the 5 digit system is simple and more suitable for describing combat injuries [49]. Additionally, the MCIS is highly correlated to the combat capability of the wounded when returning to the battlefield and can better reveal the combat capability of the wounded after injury [49].

#### Survival assessment

Currently, the commonly employed survival probability rating systems include the Trauma and Injury Severity Score (TRISS) and A Severity Characterization of Trauma (ASCOT) [50, 51]. The equation used in TRISS is $Ps=1/\left(1+{e}^{-b}\right),$where *b* = *b*_0_ + *b*_*1*_ × (*RTS*) + *b*_*2*_ × (*ISS*) + *b*_*3*_ × (*A*) and *e* = 2.718282; *RTS* is the corrected trauma score, ISS is the injury severity score, A is an age parameter (A = 0 for ages < 55 years and A = 1 for ages > 55 years), and the weights of b_0_, b_1_, b_2_ and b_3_ are different in the different injury mechanisms for blunt and penetrating wounds. ASCOT combines the physical indices, anatomical indices, age, and injury type in the given score. The characteristic of ASCOT is the further detailing of the age score. Furthermore, for the anatomical index, ASCOT adapted anatomical point scores based on the AIS-85 and thus overcame the disadvantage of the ISS in the lack of consideration for multiple organ injuries. These two methods can also be implemented to assess survival of the wounded. However, the AIS score should be replaced with the military AIS score.
